# Hemodialysis interval and its association with emergency care and mortality: A nationwide population-based cohort study

**DOI:** 10.1097/MD.0000000000014816

**Published:** 2019-03-08

**Authors:** Ching-Wen Chien, Chi-Jung Huang, Zi-Hao Chao, Song-Kong Huang, Pei-En Chen, Tao-Hsin Tung

**Affiliations:** a Institute for Hospital Management, Tsing Hua University, Shenzhen Campus, China; b Institute of Hospital and Health Care Administration, National Yang-Ming University, Taipei; c Faculty of Public Health, College of Medicine, Fu Jen Catholic University, New Taipei; d Association of Health Industry Management and Development; e Department of Medical Research and Education, Cheng Hsin General Hospital, Taipei, Taiwan.

**Keywords:** chronic renal failure, end-stage renal disease, hemodialysis, interdialytic interval

## Abstract

End-stage renal disease (ESRD) incidence in Taiwan is highest worldwide. This study analyzed the relationship between health resource use and patients on hemodialysis (HD) asking for medical help as well as the outcomes in Taiwan.

This was a retrospective cohort study that analyzed the medical data of patients on dialysis, which were collected from the National Health Insurance Database of Taiwan for the period 2000 to 2010. The study sample was screened out, and new patients starting HD from 2001 to 2005 were considered.

The daily distribution of patients with ESRD in the Monday, Wednesday, and Friday (MWF) and Tuesday, Thursday, and Saturday (TTS) groups who underwent emergent HD showed remarkable person–time on Monday and Tuesday, respectively. The disease (complication) distribution in the MWF group was higher than that in the TTS group, and the statistics of heart-failure-associated diseases were significantly different. Considering 5-year survival status, the mortality rate of patients with HD was 21.94% (255 of 1162), among which those with a history of cerebrovascular disease and diabetes were 68.63% and 72.16%, respectively.

Long interdialytic intervals may induce emergency dialysis. Therefore, the frequency of emergent HD therapy has increased (thrice a week), as predicted in the current HD policy.

## 1 Introduction

Chronic kidney disease (CKD) and its related health and economic burdens are a crucial concern for global public health.^[1]^ End-stage renal disease (ESRD) incidence in Taiwan is once the highest in the world. In 2015, the National Health Insurance (NHI) Administration of the Ministry of Health and Welfare indicated that more than 70,000 Taiwanese citizens currently had chronic renal failure. In the Taiwanese health care system, hemodialysis (HD) is generally conducted once every 2 days, thrice a week (Monday, Wednesday, and Friday or Tuesday, Thursday, and Saturday). Because Taiwanese hospitals do not conduct conventional dialysis on Sunday, patients receiving HD on Friday or Saturday must wait 3 days before their subsequent treatment on Monday or Tuesday. If patients undergoing HD have other cardiovascular diseases or receive dialysis after an excessively long interval, increases in uremic toxins and poor water removal may lead to emergency medical treatment, and patients may experience acute myocardial infarctions or strokes. Compared with healthy individuals, patients on long-term dialysis have a higher risk of developing atherosclerosis and experiencing both ischemic and hemorrhagic strokes.^[2–6]^ Mortality risk is significantly high (approximately 50%) among patients on dialysis and requiring in-patient emergency medical treatment.^[7]^

Once Taiwanese patients are diagnosed with irreversible uremia by a physician after comprehensive assessment, they must submit an application for proof of catastrophic illness to the agency of the NHI Bureau specializing in chronic renal failure before beginning the regular dialysis treatment. When the interval between dialysis treatments reaches 3 days (from Friday to Monday or Saturday to Tuesday), patients on HD are susceptible to pleural effusion, electrolyte imbalances, and excessive levels of nephrotoxins and must seek medical treatment as soon as possible. When necessary, patients must receive emergency dialysis treatment (removing excessive water and toxins).^[4,6,7]^ In Taiwan, medical expenses related to chronic renal failure are higher than those for any other disease, totaling approximately NT$45.3 billion annually. The use of emergency medical resources by patients on HD may also result in the consumption of NHI resources, preventing them from being appropriately allocated to patients with other illnesses. Furthermore, the increase in patients on dialysis requiring emergency care places additional stress on emergency room resources.^[8–10]^

Literature has shown that patients needing long-term HD with longer intervals between dialysis treatments have higher mortality rates, particularly for mortality associated with heart failure; they also have higher rates of hospitalization related to cardiovascular diseases.^[11–16]^ However, the relationship between dialysis schedules and receiving emergency dialysis has not been investigated extensively. Therefore, this study used the Taiwanese NHI database information to examine whether an extension of time between dialysis treatments causes an emergency utilization of NHI resources and further mortality.

## 2 Methods

### 2.1 Database

**Figure 1:**
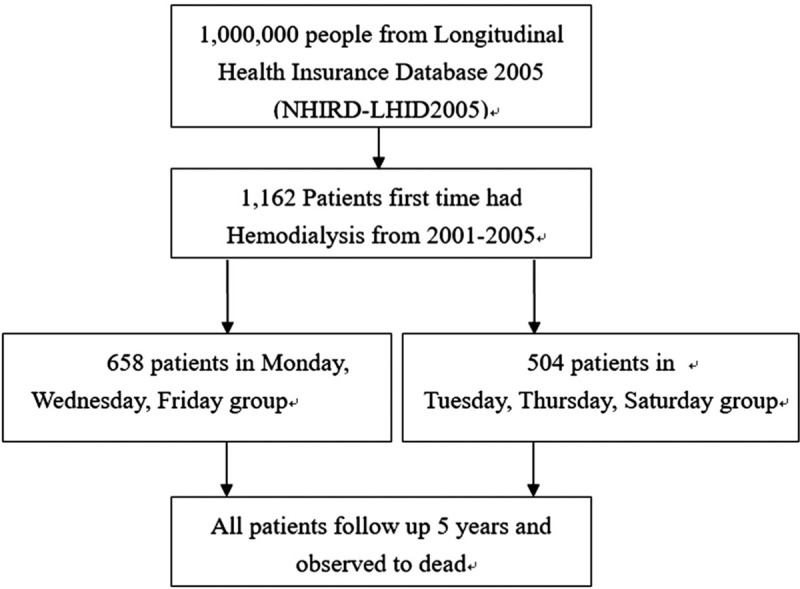
Flow chart of selection of the study population.

This was a retrospective cohort follow-up study that used the 2005 coverage file as the basis for a million-person sample collected from NHI data from 2000 to 2010 on patient health insurance claims and utilization of medical resources. The sampling process is shown in Figure 1. The enrollees of this study were patients diagnosed for the first time with chronic renal failure (International Statistical Classification of Diseases, 9th Revision, code: 585) between 2001 and 2005 who regularly received HD (NHI code: 58001C, 58027C, and 58029C) >10 times per month over a duration of ≥3 months. The participants were categorized by their regular dialysis schedules (Monday, Wednesday, Friday [MWF] as one group; Tuesday, Thursday, Saturday [TTS] as the other), and patients who changed their dialysis schedule or treatment location were excluded from the study. Patients were then observed over the next 5 years (2006–2010) to examine trends related to emergency treatment and death. The National Health Research Institute had assured confidentiality; therefore, all procedures were performed in accordance with the guidelines of our institutional ethics committee and adhered to the Declaration of Helsinki. Because the data source was in the public domain and anonymized, informed consent was not given. This study was exempted from review by the Institutional Review Board of Taipei Veterans General Hospital (IRB-TPEVGH No: 2015-11-001BC).

### 2.2 Study variables

This study evaluated differences in the incidence of emergency dialysis and death between 2 groups of newly diagnosed patients regularly receiving dialysis. Basic patient variables were categorized into personal and hospital dimensions, with the hospital dimension comprising ownership (public or private), teaching status, and hospital accreditation level (medical center, regional hospital, district hospital, or primary care clinic). The personal dimension comprised sex, age, and past medical history (cerebrovascular disease, ischemic heart disease, heart failure, arrhythmia, arterial disease, other types of heart disease, chronic obstructive pulmonary disease, gastrointestinal bleeding, liver disease, cancer, and diabetes). This study investigated the effect of these dimensions on the incidence of emergency dialysis and death for newly diagnosed patients on dialysis belonging to the 2 different time schedule groups.

### 2.3 Statistical analysis

This study used SAS 9.3 for data compilation and statistical analysis. For descriptive statistics, the variable properties were divided into categorical variables and continuous variables. Usage frequency and percentage were used to express the distribution of categorical variables, whereas means and standard deviations were used to express the distribution of continuous variables. For inferential statistics, this study conducted univariate analysis to examine the correlations between each variable. Chi-square testing was used for correlation analysis of different groups and their personal dimension variables (i.e., sex, urbanization, comorbidity status, emergency dialysis incidence, and death status) as well as hospital dimension variables (i.e., accreditation, ownership, and teaching status). An independent-sample *t* test was used to examine the age distribution of the different groups and whether the 2 groups subsequently exhibited differing mortality rates. Lastly, this study used the Cox regression model for multivariate analysis to examine the effect of dialysis grouping on patient mortality after controlling correlated factors. The level of significance adopted for this study was *P* < .05.

## 3 Results

**Figure 2:**
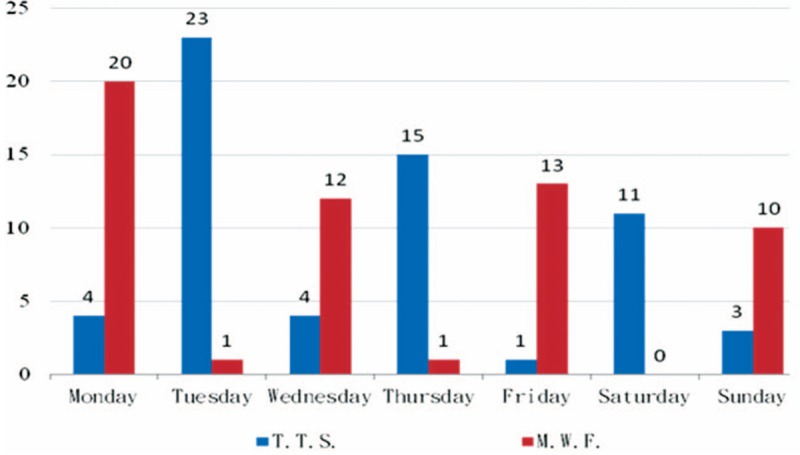
The patients’ visits emergency dialysis cumulative count between Monday-Wednesday-Friday (MWF) and Tuesday-Thursday-Saturday (TTS).

As illustrated in Figure 2, emergency dialysis for both patient groups was administered most frequently on Mondays (24 cases) and Tuesdays (24 cases). The MWF group sought emergency treatment most frequently on Mondays (*P* = .008), whereas the TTS group sought emergency treatment most frequently on Tuesdays (*P* < .0001).

The distributions of basic variables and personal dimension variables for newly diagnosed patients undergoing dialysis are listed and categorized by their regular dialysis schedule in Table 1. Although the distributions of sex and age between the 2 groups differed, with the MWF group exhibiting a higher sex ratio and the TTS group exhibiting a higher average age, the difference between the 2 was nonsignificant. For the hospital dimension variables, the distribution of teaching status and hospital ownership did not reveal any significant difference; however, the majority of the patients received treatment at teaching hospitals and private hospitals. However, the hospital accreditation variable exhibited a significant difference between the 2 groups, with the MWF group exhibiting a higher distribution. Regarding the primary observation results, the difference in the distribution of emergency treatment or death between the 2 groups was nonsignificant.

**Table 1:**
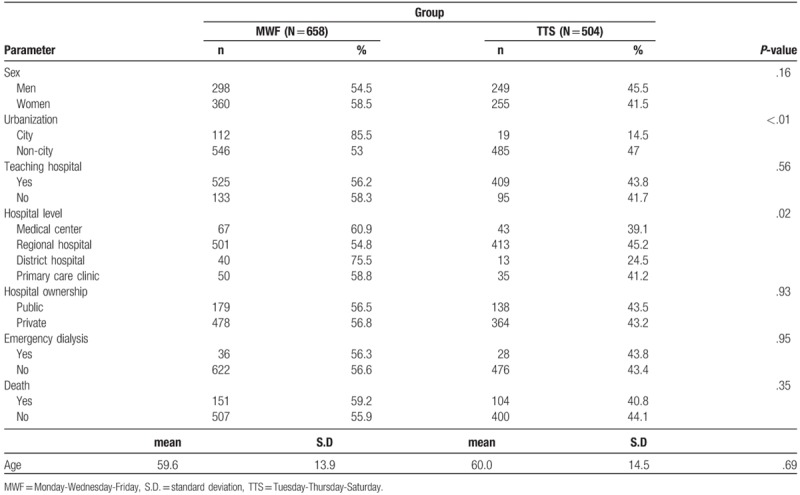
The basic demographic characteristics of the study population (n = 1162).

Table 2 outlines the comorbidity variable distribution of the 2 groups of patients on dialysis examined in this study. The comorbidity distribution of the MWF group was higher than that of the TTS group. However, only heart failure reached significance; the distribution of all other related comorbidities was nonsignificant.

**Table 2:**
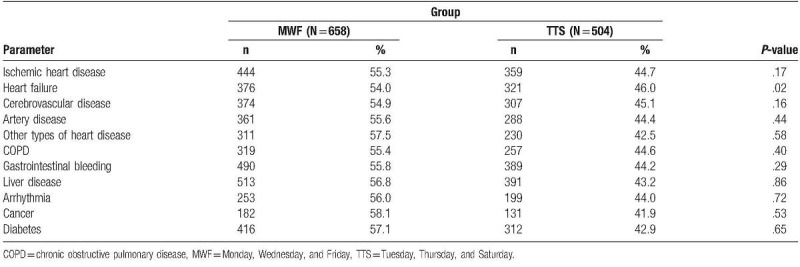
The comorbidity of the study population (n = 1162).

Table 3 lists the effects of different variables on the patients examined in this study as well as their 5-year survival rates. After correction for confounding variables, the mortality risk for the MWF group was determined to be 1.21 times that of the TTS group (95% confidence interval [CI]: 0.94–1.57). Although the risk was comparatively higher, the difference in the actual incidences of death between the 2 groups was nonsignificant in the 5-year tracking period. Furthermore, the mortality risk of those receiving emergency dialysis was 0.94 times that of those not receiving emergency dialysis (95% CI: 0.52–1.70; *P* > .05). Regarding personal dimension variables, the mortality risk of men was 1.07 times that of women (95% CI: 0.83–1.38; *P* > .05). The older the patients were, the higher was the mortality risk, with a risk ratio of 1.04 (95% CI: 1.03–1.06); the result for this variable reached statistical significance. For hospital variables, this study used medical centers, the highest category of hospitals, as the baseline. Regional hospitals, district hospitals, and clinics exhibited a patient mortality risk of 1.01 (95% CI: 0.65–1.58), 0.68 (95% CI: 0.30–1.54), and 0.64 (95% CI: 0.29–1.42) times baseline, respectively; none were statistically significant. The mortality risk exhibited by public hospitals was 0.96 times that of private hospitals (95% CI: 0.73–1.26) and was not statistically significant. The risk of patient mortality for teaching hospitals was 1.87 times that of nonteaching hospitals (95% CI: 1.22–2.87), reaching a level of statistical significance. In addition, for comorbidities, mortality risk for patients on dialysis exhibiting gastrointestinal bleeding was 1.77 times that of patients without this symptom (95% CI: 1.24–2.54; *P* < .05). Furthermore, the mortality risk for patients on dialysis with arterial disease was 0.59 times that of those without this symptom (95% CI: 0.46–0.76; *P* < .05). The remaining comorbidities were not statistically significant.

**Table 3:**
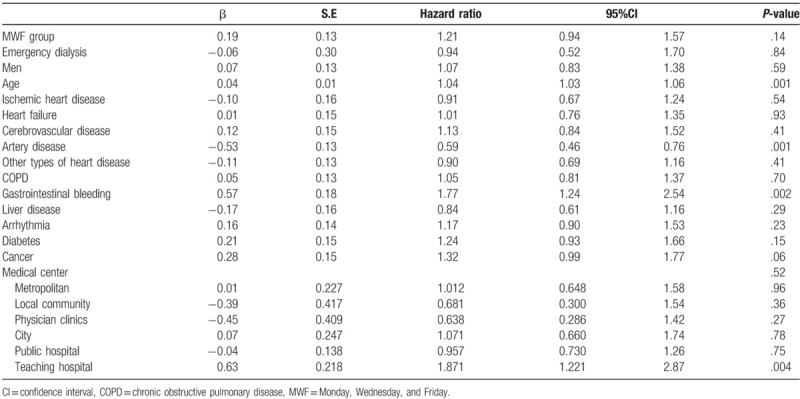
The hazard ratio of each variable by Cox proportional hazard regression model.

## 4 Discussion

### 4.1 Clinical implications

Analysis of the 5-year mortality of patients on HD indicated that 255 of 1162 (21.94%) patients died during the follow-up period. Of the patients who died, 68.63% had cerebrovascular disease, 72.16% had diabetes, 85.49% experienced gastrointestinal bleeding, and 65.49% had heart failure. Previous studies indicated that the incidence of acute cardiovascular disease, in particular the incidence of sudden death from cardiovascular causes, is higher on Mondays than on other days, with the majority patients experiencing this aged >65 years. In addition, atrial fibrillation, diabetes, hyperkalemia, and HD methods affected the likelihood of death for patients receiving HD.^[17]^

The sample population used in this study consisted of patients with newly diagnosed chronic renal failure and indicated that the frequency of receiving emergency dialysis was higher on Monday (MWF group) and Tuesday (TTS group) compared with the other days of the week. Patients having other comorbidities were more likely to require emergency dialysis. The results implied that the higher frequency of emergency dialysis on Mondays and Tuesdays was primarily related to patients’ cardiovascular disease. However, because this study did not have access to data on cause of death, further comprehensive statistical analysis of comorbidity data of long-term mortality is required.

A comparison of dialysis frequencies of 6 times per week and 3 times per week revealed that frequent HD is related to controlling hypertension and hyperphosphatemia.^[18]^ Frequent HD (more than 3 times per week) improves the quality of life and health of patients with chronic renal failure, potentially increases their lifespan, and may reduce the likelihood of hospitalization and the occurrence of comorbidities. Although standard dialysis treatment effectively enables the majority of patients with chronic renal failure to maintain their daily living activities, it cannot restore such patients to their previous health. These patients may also have higher rates of hospitalization because of comorbidities.^[19]^ In addition, frequent HD treatment exhibits a higher cost-effectiveness than standard HD. As such, administering HD more frequently may affect dialysis costs.^[19,20]^ Because daily dialysis equates to a higher treatment volume and corresponding dialysis costs, patients may experience lower incidence rates and corresponding medication costs as well as lower hospitalization expenses.^[21]^ One study examined patients receiving daily dialysis treatments either during the day or night and reported that the difference between the two patient groups regarding hospitalization rates and duration was nonsignificant.^[22]^ The difference between the study and control groups in terms of the annual emergency treatment rates and vascular access was nonsignificant.^[22]^

Bleyer et al indicated that, after categorizing patients into MWF and TTS groups and examining the ratio of people with sudden cardiac death (SCD) throughout the week, the MWF group exhibited a higher rate of SCD on Mondays, whereas the TTS group exhibited a higher rate on Tuesdays. Related factors were identified as excessive patient water retention and increases in blood potassium causing pleural effusion and hyperkalemia, both causes of SCD.^[23]^ Wong et al indicated that patients with chronic renal failure receiving HD are at a high risk for SCD. The primary causes of SCD are severe bradycardia and cardiac arrest, with an extended interdialytic period (IDP) being a possible risk factor. Typical HD (thrice per week) is composed of short IDPs of 48 h and a long IDP (LIDP) of 72 h.^[24]^ The risk of SCD and arrhythmia is most significant during the LIDP.^[24]^ Fotheringham et al indicated that patients on HD may have a higher rate of hospitalization and mortality risk on the third day after dialysis treatment because of excessive buildup of fluids, a finding that may be strongly related to the higher incidence of SCD among patients receiving HD.^[25]^ Education of patients and dietary control have significant positive effects on blood pressure. Therefore, patients on HD can reduce the occurrence of comorbidities through daily diet and fluid control, continual nutritional education, and regular evaluations.^[26,27]^ The provision of patient health education, particularly regarding the limiting of fluid intake in the diet, with clinical medical teams is instrumental. Particularly useful is the prevention of careless dietary moderation during the 3 days over the weekend (Saturday and Sunday) until the dialysis treatment, as well as during long consecutive holidays, to avoid excessive water retention. Hecking et al indicated that interdialytic weight gain between HD treatments is associated with chronic fluid overload and a high mortality risk.^[28]^ For adults on HD, folic acid supplementation may reduce mortality from cardiovascular disease and other causes.^[29]^

In this study, all-cause mortality was not different between TTS and MWF groups, and emergency department services occurred more frequently in the last day of interdialytic interval. Within each group, a similar pattern of emergency department services occurrence as a function of time after dialysis was showed. Our results of higher emergency dialysis after the long interdialytic interval is impressive. The cause of this “post weekend” effect is essential that how in-center dialysis treatments are structured.^[30,31]^ In Taiwan, in-center hemodialysis patients either of MWF or TTS group, the between-dialysis (interdialytic) interval is 48 h except for the Friday to Monday and Saturday to Tuesday intervals which are experienced as 72 h. It is possible that the longer 72 h interdialytic interval may lead to progressive fluid accumulation, more severe electrolyte derangements, higher cardiovascular instability, and resulting in higher need for emergent care or death on the last day (Monday or Tuesday) of the time interval.^[30]^ From the clinical viewpoint, whether the increased risk for adversity following the long interdialytic interval applies to all dialysis patients or is only restricted to a subset, is an issue of critical essential and should need to be further addressed in future long-term follow-up studies. The importance is that while strategies exist to eliminate the long dialytic interval, such as short daily hemodialysis and home hemodialysis, these strategies have implications in terms of patient medical care burden and cost to payers. The economics from patient, society, and payer perspectives will also need to be better explored and balanced before clear treatment recommendations could be made.

### 4.2 Methodological considerations

There are a few limitations in this study. Firstly, the NHIRD dataset does not contain detailed information regarding socioeconomic status and family history of diseases, all of which may be risk factors for emergency care and mortality. Secondly, the evidence derived from a retrospective cohort study is generally lower in statistical quality than that from randomized trials because of potential biases related to adjustments for confounding variables. Thirdly, the meticulous study design and control measures for confounding factors was used, bias resulting from unknown confounders may still have affected these results. Fourthly, all data in the NHIRD are anonymous, that is, relevant clinical variables, such as serum laboratory data, imaging results, and pathology findings were unavailable regarding the study patient cases. Fifthly, lack of access to death causes in studied patients may hinder the study conclusions. Sixthly, because our results could not differentiate among patients who received dialysis during the morning, afternoon, and evening, groups were categorized based on dialysis treatment dates. As such, this study was unable to clearly analyze differences between each group on the basis of treatment time. In the future, the range of data should be reduced by, for example, analyzing the patients of only one hospital. Finally, ethnicity and in- or out-hospital mortality may also add potential bias.

## 5 Conclusion

Long interdialytic intervals may induce emergency dialysis. This has increased the frequency of emergent HD therapy, which is predicted in the current HD policy (thrice a week).

## Author contributions

**Conceptualization:** Chi-Jung Huang, Song-Kong Huang, Tao-Hsin Tung.

**Data curation:** Zi-Hao Chao.

**Methodology:** Chi-Jung Huang, Zi-Hao Chao, Pei-En Chen.

**Project administration:** Song-Kong Huang.

**Supervision:** Song-Kong Huang.

**Writing – original draft:** Chi-Jung Huang.

**Writing – review & editing:** Ching-Wen Chien, Tao-Hsin Tung.
